# Hospital Based Quality of Life in Oral Cancer Surgery

**DOI:** 10.3390/cancers12082152

**Published:** 2020-08-04

**Authors:** Carolin Goetz, Julius Raschka, Klaus-Dietrich Wolff, Andreas Kolk, Oliver Bissinger

**Affiliations:** 1 Department of Oral and Maxillofacial Surgery, Technische Universitaet München, Fakultaet für Medizin, Klinikum rechts der Isar, Ismaninger Str. 22, 81675 Munich, Germany; cg.goetz@tum.de (C.G.); J.Raschka@campus.lmu.de (J.R.); klaus-dietrich.wolff@tum.de (K.-D.W.); andreas.kolk@i-med.ac.at (A.K.); 2 Department of Oral and Maxillofacial Surgery, Medizinische Universitaet Innsbruck, Anichstraße 35, 6020 Innsbruck, Austria

**Keywords:** EORTC QLQ-C30, H&N-35, oral cancer surgery, quality of life, in-patient stay, malfunctions, postsurgery needs

## Abstract

The diagnosis of cancer and its treatment have an incomparable impact on a patient’s life. In the early postoperative stages after the surgical treatment of oral squamous cell carcinoma (OSCC), functions and well-being are limited, which leads to a fundamental decline of the quality of life (QoL). To date, no studies have been performed that focus on the development of special aspects during the time of the in-patient stay of OSCC patients. With the results of this cross-sectional study, we are able to identify those patients who tend to require special support. This cross-sectional study determined the postoperative QoL with a questionnaire (QU) that was handed out twice to OSCC patients after surgery during their inpatient stay. The questions were based on the European Organisation for Research and Treatment of Cancer (EORTC)’s Quality of Life Questionnaire (QLQ)-C30 and QLQ-H&N35. In our study, we found that for postoperative OSCC patients, eating, swallowing and speech were influenced the most. After decannulation, tracheotomy showed no impact on functions. Social contact was impaired at both timepoints. Especially female patients consider themselves to be more impaired on the scale of social contact. QoL should be checked with a standardized QU as an established tool during hospitalization in every oncology department. Only this procedure can pinpoint those patients who have struggles with their surgical outcome and need more assistance.

## 1. Introduction

The diagnosis of cancer has an incomparable impact on a patient’s life, their quality of life, and also raises a wide variety of issues [1]. The sixth most common cancer worldwide is oral squamous cell carcinoma (OSCC), which is often caused by smoking and alcohol consumption [2]. It is nowadays well studied, and the infection of Human papillomavirus (HPV) is considered to play a minor role in OSCC [3]. The detection of predictive biomarkers is the aim of many ongoing studies, especially inflammatory crosstalks and the extent of distinct inflammation molecules, which are recent topics with great capability. More and more results show that the mechanisms seen in periodontitis are important for other diseases, such as coronary heart disease and cancer [4]. OSCC, in particular, frequently affects several diverse structures and can lead to symptoms such as dysphagia, hoarseness and otalgia, even during their early stages of development [5]. With regard to treatment possibilities, surgery is considered the best option according to several international guidelines of oral cancer (OC) [6]. Reconstruction of the oral anatomy with microvascular flaps leads to the best postoperative results with a high quality of rebuilt functions such as breathing, swallowing, and food intake, as well as providing good aesthetics and dental rehabilitation for the patients [7]. The patients therefore experience a good quality of life (QoL) [1]. Nevertheless, in the early postoperative stages after the surgical treatment of OSCC, the above-mentioned functions can be limited [8,9]. This is despite the extended preoperative informational talks about the procedure and the upcoming postoperative situation that patients are sometimes confronted with in their postoperative situation. This can lead to a fundamental decline of their QoL, especially in the early postoperative stages [8]. Patients are extremely vulnerable during this period, as they have new needs closely dependent upon their new circumstances.

In order to measure QoL, the European Organisation for Research and Treatment of Cancer (EORTC) developed a Quality of Life Questionnaire Core-30 (EORTC QLQ-C30) in conjunction with module H&N-35; this has become a highly tested measure with a large body of evidence ensuring reliability and validity [10,11,12,13]. These questionnaires (QUs) include all head and neck cancers [12]. QoL is a multi-dimensional construct [14,15,16] and the diverse cancer locations lead to different impaired dimensions of QoL. Therefore, we have designed a QU specialized for OSCC with a shortened version of the original to maintain compliance during the in-patient stay. Several studies have revealed that a more differentiated view concerning the cancer location is desirable with regard to QoL [17]. Whereas a recent study confirms that QoL in OSCC returns to baseline levels at one year after microvascular reconstruction [18], another study has shown that long-term QoL is significantly reduced in the dimensions of speech [19]. The rational of the study was to evaluate: (1) to what extent QoL is reduced immediately after surgery, (2) the functions and symptoms that are affected during the early stages, (3) the extent of these changes to the QoL and (4) the way that these aspects change during the in-patient stay. Our null hypothesis was that patients are affected most in the early period of the inpatient stay and improve their situation during the stay. Further, to date, no studies have been performed that focus on the development of these aspects during the time of the in-patient stay. With the results of this cross-sectional study, we are now able to improve our support for the patients and to identify those patients who tend to require special support.

## 2. Material and Methods

### 2.1. Study Design

All included patients suffered from OSCC and were treated surgically during the year 2019 at the Department of Oral and Maxillofacial Surgery, Klinikum rechts der Isar, Technische Universitaet Muenchen, Germany. The Ethics commission of the Technische Universitaet Muenchen gave full approval of the study. The reference number is 275/19 S. This cross-sectional study was conducted according to the declaration of Helsinki. Consent for publication was obtained by every patient by a signed written form. The datasets during and/or analyzed during the current study are available from the corresponding author on reasonable request. QoL was evaluated by using two questionnaires (QU) (see Appendix A) based on EORTC-QLQ-C30 Version 3.0 and its supplement, H&N35. These QU were designed to delineate precisely on the dimensions of QoL in OSCC patients and were assessed in a similar manner to the QU of the EORTC [20]. They included 4 function and 16 symptom scales. The assignment of the questions to the various scales is shown in Table 1. As described in the EORTC QLQ-C30 and EORTC QLQ H&N-35 scoring manuals [20], the raw scores of the QU were transformed into scales of 0–100. A high score implied a high level of functioning but, in contrast to other QU, a high score in the symptom scales indicated a low level of symptoms or problems. Thus, a high score represented a positive outcome in all scales. Patients answered the first QU the day after decannulation. In cases where no tracheotomy took place, we gave the first QU at the second day after surgery. The second QU was answered the day they were discharged from hospital. Out of 36 patients who completed the first QU, 26 patients completed the second QU and were eligible for evaluation. Ten patients have been excluded because they were still cannulated and therefore not able to speak or not interested in taking part in further questionnaires.

### 2.2. Data Analysis

In addition to the two QU, the following data were collected: basic patient data such as age, gender, tumor location, TNM (tumor, nodes, metastasis) stage, type of microvascular flap reconstruction and comorbidities. The data were listed and processed in Microsoft Excel (Microsoft, Version 1908, Redmond, QA, USA) for descriptive statistics. Within Microsoft Excel, the Wilcoxon signed rank test was used for the analysis of score differences at both timepoints. Moreover, patients were divided into groups for each TNM classification, if tracheotomy or neck dissection was performed, and gender. The Mann–Whitney U Test, also performed in Microsoft Excel, was used to analyze the score between the two groups. Data were considered statistically significant for *p*-values smaller than 0.05.

## 3. Results

### 3.1. Patient-Specific Data

Of the 26 patients included in this study, 14 (54%) were female and 12 (46%) were male. The mean age was 67 years (range 31–85 years). During the first survey, 17 (65%) patients had a nasogastral feeding tube and one patient had a percutaneous endoscopic gastrostomy (PEG) tube. Three patients had a PEG at the time of the second QU and left hospital with a PEG-tube (for further patient-specific information, see Table 2).

All patients received primary reconstruction: 23 patients with a microvascular flap, and three patients with a local flap (for further treatment-specific information, see Table 2).

### 3.2. Quality of Life Assessment

Most patients answered the first QU six days (median: 6, range 4–11) and the second QU 10 days (median 10, range 8–23) after surgery. For the first QU it was not possible to interview the patients at a specific day after surgery because of prolonged postoperative intensive care, postoperative delirium and remaining cannulated after tracheotomy for a longer period of time. We set it on that day, when the cannula was removed, and in cases of surgery without tracheotomy, we set day 2 after surgery for the day for QU I. The second QU depended on the day patients were discharged from hospital and therefore differs regarding which point in time. This implies that the median time of stay in hospital is 11 days because, in 23 cases, patients answered the second QU on the last day before leaving the hospital. Table 3 shows the median scores of all collected scales with interquartile ranges for both QUs. Some scales showed a score of 100 and therefore were minimally influenced on QoL scales at six days after surgery and when the patient left hospital. Table 3 shows the median scores of all collected scales with interquartile ranges for both QUs. Some scales showed a score of 100 and therefore were minimally influenced on QoL on the first survey and on the second survey. No problems were experienced with nausea and vomiting. Moreover, no measurable constipation or diarrhea was noted. Although the median of cognitive functioning, pain, dyspnea and problems with the senses was 100, some patients had problems with these issues, as can be seen in the interquartile range differences up to 50 score points. However, in QU I, 23 (88%), and in QU II, all included patients, stated that they received sufficient pain therapy.

The overall highest impairment involved speech problems, with a median in score of 25, followed by role functioning. Furthermore, almost no differences were found between the first and second QU with regard to these scales. The median of speech problems did not change and, although the median of role functioning jumped from 25 to 50, no difference was seen in the interquartile range (IQR). Furthermore, the score of insomnia decreased to a median of 38 and that of fatigue to a median of 50. However, additional questioning revealed that sleep was strongly influenced by the in-patient stay. The patients stated, while answering the QUs, that their sleep was interrupted by the patient sharing their room and by nurses entering the room at night in order to carry out checks. Social contact problems were impaired as reflected by the score of 75, but no measurable differences were apparent between the two QUs.

Nevertheless, two scales improved significantly during the in-patient stay. On the one hand, problems with swallowing improved to a median score of 56 at the time of the second QU. This also means that the patients were still experiencing problems with regard to swallowing when they left hospital. On the other hand, physical functioning improved to a median score of 100 at QU II from a score of 67 at QU I. The Wilcoxon signed rank test showed a difference concerning the aspects of swallowing and physical functioning between both QUs with a *p*-value of <0.001.

Importantly, we need to mention once again that 17 patients (65%) had a nasogastral feeding tube (see Table 4) when they answered the first QU. Therefore, questions regarding problems with swallowing were difficult for them to answer. Moreover, some cases were counted as experiencing severe problems with swallowing as they were not allowed nutrition (fluid, soft or solid food) by oral intake and had a nasogastral feeding tube following the recommendations of the speech therapy consultant.

Table 5, Table 6 and Table 7 list some selected group comparisons that show promising disparities between the various groups.

Table 5 shows the median and IQR of selected QoL scales with respect to the tumor size (T-score) of the patients. With regard to the function scales, minor differences are apparent in the first QU. In particular, concerning the scale physical functioning, patients in T1–T2 stage had a higher score (median of 67) than patients with T3–T4 stage (median of 42). The median of insomnia also showed a difference: T1–T2 stage patients had a median score of 63 and patients with T3–T4 stage had a median of 25, while the IQR was widely spread. With regard to problems with swallowing, in the second QU, patients with a T1–T2 stage had a median score of 72 compared with the group of T3–T4 stage tumors, which had a median score of only 38. Similar findings were found for the aspect of dry mouth during the second QU.

In Table 6, the collective data were split depending upon whether tracheotomy was performed during surgery. Of importance, decannulation was done and the tracheal tube was no longer present at QU I or QU II. The median scores regarding insomnia in patients with a tracheotomy were 25 points lower in both QUs than for patients without a tracheotomy. However, surprisingly, no changes were observed in the scores concerning the scales for swallowing and speech problems.

With respect to gender, Table 7 shows differences between female and male patients in both QUs. Whereas the median score of insomnia was almost steady at 63 for male patients, female patients had a lower score of 25 in the first QU. Moreover, differences were measurable for social contact. Male patients felt almost no impairment in this regard (the median score was 100 in the first QU and 81 in the second QU) but female patients had a lower median (56 and 63) and IQR. However, the Mann–Whitney U test showed no significant differences between each of the two groups in Table 5, Table 6 and Table 7.

## 4. Discussion

In this cross-sectional study, QoL was assessed by using two QUs based on the EORTC QLQ-C30 and EORTC QLQ H&N-35 at two different timepoints after surgery for OSCC patients. The aim of this study was to evaluate QoL early after surgery and by the end of hospitalization. As EORTC QLQ C30 and H&N-35 QU were developed to evaluate QoL in all head and neck cancers without specification as to the localization [12], this study used a QU that was developed specifically for the measurement of QoL in patients with OSCC. Further, a shorter QU, such as the H&N-35 QU, was used to increase compliance caused by the shorter duration of answering the QU. The results of this study show that some QoL scales are little influenced by the surgical intervention. At the first timepoint (QU I), patients felt no nausea and had no problems with diarrhea and constipation. Furthermore, pain therapy with non-steroidal anti-inflammatory drugs seemed to be sufficient for the patients and, therefore, they complained of no general and local pain. Because of their lack of pain and because the following issues influence social integration the strongest [21], other aspects such as speech, chewing and swallowing became more important than pain for patient QoL [22]. Our null hypothesis was approved, since we saw the increase in QoL during the in-patient stay.

Surgery for OSCC affects many structures that are necessary for adequate articulation [23]. Our patients felt most impaired in speech and almost no difference was measurable between QU I and II. Surprisingly, as can be seen in Table 6, tracheotomy had a minor influence on speech problems in our cohort, with patients without tracheotomy having similar scores on this scale. Patients were given QU I when they had been decannulated, but they felt only minor improvement at QU II. Yet, it is not discussed in literature since other studies were not conducted during the in-patient stay. Our data further show that patients had minor problems with phonation and severe problems with articulation. Speech needs the vocal signal to be audible and needs the organs of the vocal tract to shape the speech sound by articulation. At QU II, patients had less problems producing an audible sound than articulating intelligibility [24]. However, these findings were not measured in any kind. Speech was also much influenced by the tumor stage, with there being less impairment in patients with smaller tumor sizes (T1–T2 stages) because relevant structures were less affected, and the resected area was minor. This can also be explained by the need for smaller grafts to reconstruct the resection side. The finding, that the intelligibility of speech significantly decreases with the increase in tumor size is also seen in literature [25,26].

Role functioning was also impaired greatly in both QU. In addition, tumor stage 3 and 4 shows minor differences in median score and IQR. As the measurement of the role functioning of patients in hospital is complicated, the created QU included only one question concerning this scale, in contrast to the EORTC C30 questionnaire containing two questions in this regard. Therefore, role functioning was of less concern in this study. The patients also suffered from insomnia and fatigue. However, as stated above, the sleep of patients was strongly influenced by the in-patient stay. Therefore, no conclusion could be drawn as to whether their insomnia and fatigue were related to their disease and postoperative condition or to their stay in hospital. Nevertheless, gender-specific differences were identifiable. Although these findings are not significant (Table 7), further research might reveal ways to improve the sleep of patients in hospital and therefore to increase patient recovery and QoL.

A significant improvement was apparent concerning swallowing problems between QU I and QU II. Although surgery for OSCC obviously affects the swallowing function, this improvement might be explained by postoperative swelling, which declines during the in-patient stay. QU I was given mostly on the sixth day after treatment to the patients, the reduction in swelling might strongly influence swallowing in a positive way and lead to a much higher score in QU II. However, patients were provided with a nasogastral feeding tube intraoperatively in order to provide nutrition and to prevent any disturbances in healing. During the second survey, 17 patients still had a nasogastral feeding tube. Linked to this, it is important to evaluate the nutrition situation of the patient, since malnutrition is affecting the prognosis and also the QoL, as seen before in other studies and other morbidities [27]. Therefore, the scores concerning problems with swallowing during the first QU might not be reliable as, in these cases, swallowing problems could not be measured. Nevertheless, advanced tumor stages showed lower QoL scores for swallowing in the second QU, as has been shown in a previous study [28]. Tracheotomy, however, showed no effects on swallowing after decannulation.

With regard to physical functioning, this scale was not as much affected by the surgery as was swallowing. Nevertheless, the Wilcoxon test showed a significant improvement in scores between QU I and QU II. This improvement is probably explained on the basis of the reacquired mobility at the time of QU II and, thus, the patient having the ability to wash and dress themselves and eat without help. Moreover, a difference can also be seen in the score with regard to tumor stage. Again, advanced tumor stages showed a poorer result in QU I. Nevertheless, during the QU II, this score also returns almost to baseline in this group.

The scale for dry mouth also showed poorer values and did not change between QU I and QU II. Indeed, the scale for dry mouth also receives poor scores in long-term QoL, since radiation therapy is playing a much more significant role than surgery [29]. Moreover, T-stage (≥T3) is a significant predictor of dry mouth, since advanced tumor stages are more often getting postoperative radiation therapy [30]. Table 5 further reveals differences between the tumor stage and the complication of dry mouth, although this difference is not statistically significant.

The social contact item shows no differences between QU I and II, although social contact is impaired at both timepoints. Older and male patients tend to rate their appearance more positively [31,32]. This can partly be seen in Table 7, where female patients consider themselves to be more impaired on the scale of social contact. In our QU, we reduced this scale from four to two scales in comparison with the H&N-35. Two of the omitted items were removed from the QU, because they were not meaningful for hospitalized patients.

Overall, these findings help to understand the feelings and impairments of patients after surgery of OSCC and therefore build a foundation for increasing postoperative support. For example, a possible starting point for improvement could be to assist patients regarding sleeping problems. As stated above, patients reported that their sleep was mainly interrupted by hospital staff, which could be avoided in certain situations. On the other hand, surveillance by hospital staff is very important to ensure safe conditions. Further research in the future could provide even more starting points for improvement.

Some shortcomings exist with regard to this study. The postoperative QoL may be influenced by some other issues that we could not fully observe with the QU due to a small sample size. However, in addition, the sample size of this study is representative enough to illustrate the effects of the treatment of handing out the QU and the development of QoL scores. Furthermore, we gained the experience that the QU is inappropriate for patients that will leave the hospital with a cannula. We also see this fact as possible improvement for the QU in the future. We will design an appropriate, modified questionnaire for patients that will leave the hospital with a cannula. During the trial, we saw that many patients were not able to answer the QU for the issues mentioned above at both times. An early postoperative single-center clinical trial therefore has some sample size limitations. For this reason, we decided to see this study as a pilot study and use these findings to design a multi-center study. Further, the early postoperative setting is a situation that cannot be handled in a standardized format. On the other hand, questionnaires and surveys of this time period are lacking in literature. Therefore, we see our study as essential to obtaining further understanding of patients’ situations, to improving their situation and, hence, to continue the use of the QU. During the conductance of the study, we addressed the issues of the patients, which we gained in the first QU. This improved the situation of the patients. Moreover, as in every trial focusing on QU and QoL, it is unclear if a higher willingness to answer the QU might be influenced by whether patients experience a better or worse QoL. However, on the other hand, the QU was highly approved by the patients and, indeed, essential information is extractable also in a cohort of this size. In the future, our aim is as we did it with the help of this study, to catch the problems of the patients early at the hospital stay and to solve them during the stay. Further, we aim and to improve the reliability of the trial and popularize application of the QU. Moreover, a preoperative QoL assessment and an assessment of the QoL during follow-up appointments are planned. The present study and our conducted study of the tumor after care in OSCC will help us to solve this aim sucessfully^1^. This would also be important to compare the situation and problems of the inpatient stay to the situation of the patients during the follow-up period.

## 5. Conclusions

Eating, swallowing and speech problems are influenced the most by surgery. However, we saw an improvement in most of our OSCC patients during the in-patient stay. To our knowledge, this is the first trial to evaluate the early postoperative QoL in OSCC patients. QoL should be checked with a standardized QU as an established tool during hospitalization in every oncology department. Only this procedure can pinpoint those patients who have struggles with their surgical outcome and need more assistance. It is a procedure that should start during their stay in hospital.

## Figures and Tables

**Table 1:** Function and symptom scales.

Scale Name	Number of Items	Questions
Function scales		
Physical functioning	3	1–3
Role functioning	1	4
Emotional functioning	2	19,2
Cognitive functioning	2	11,12
Symptom scales		
Fatigue	1	10
Nausea and vomiting	2	14,15
Pain	1	6
Dyspnoea	1	5
Insomnia	1	9
Appetite loss	1	13
Constipation	1	16
Diarrhea	1	17
Local pain	3	23–25
Swallowing problems	4	26–29
Problems with the senses	2	31,32
Speech problems	1	34
Trouble with social contact	2	33,35
Dry mouth	1	30
Weight loss	1	37
Weight gain	1	36

**Table 2:** Treatment-specific information.

Variable	Specification	No. of Patients	(%)
Cancer location	Tongue	9	(35)
	Floor of mouth	7	(27)
	Jaw	5	(19)
	Buccal	4	(15)
	Lower lip	1	(4)
Microvascular flap	Radialis	11	(42)
	Fibular	4	(15)
	Soleus perforator	4	(15)
	Antero lateral tight	4	(15)
Local flap	Platysma	1	(4)
	Bernard-von Burow-von Bruns	1	(4)
	Deltopectoral flap	1	(4)
Tracheotomy	Yes	17	(65)
	No	9	(35)
Neck dissection	Yes	17	(65)
	No	9	(35)
T score	T in situ	1	(4)
	T1	7	(27)
	T2	4	(15)
	T3	6	(23)
	T4	8	(30)
Adjuvant therapy *	None	6	(23)
	Radiation	18	(69)
	Chemoradiation	2	(8)

* Recommended following an interdisciplinary tumor conference.

**Table 3:** Median score of collected function and symptom scales with interquartile range (IQR).

Scale	Questionnaire 1		Questionnaire 2	
Function Scales	Median	IQR	Median	IQR
Physical functioning	67 *	(33–90)	100 *	(79–100)
Role functioning	25	(25–75)	50	(25–75)
Emotional functioning	75	(63–100)	88	(75–100)
Cognitive functioning	100	(63–100)	100	(75–100)
Symptom Scales				
Fatigue	50	(31–100)	50	(50–100)
Nausea and vomiting	100	(100–100)	100	(100–100)
Pain	100	(56–100)	100	(50–100)
Dyspnea	100	(50–100)	100	(50–100)
Insomnia	38	(25–100)	50	(50–75)
Appetite loss	75	(50–100)	50	(50–75)
Constipation	100	(100–100)	100	(100–100)
Diarrhea	100	(100–100)	100	(100–100)
Local pain	75	(60–92)	83	(69–100)
Swallowing	25 *	(13–73)	56 *	(27–73)
Problems with the senses	100	(75–100)	100	(75–100)
Speech problems	25	(0–50)	25	(6.25–50)
Trouble with social contact	75	(50–100)	69	(50–100)
Dry mouth	50	(25–100)	50	(25–100)
Weight loss	50	(50–75)	75	(56–100)
Weight gain	100	(100–100)	100	(100–100)

* Wilcoxon signed rank test *p*-value < 0.001.

**Table 4:** Patient-specific data. PEG, percutaneous endoscopic gastrostomy; QU, questionnaire.

Parameter		Median	Range
Age in years		67.5	(31–85)
		No. of patients	(%)
Gender	Male	12	(46)
	Female	14	(54)
Nasogastral feeding tube	QU I	17	(65)
PEG	QU I	1	(4)
PEG	QU II	3	(12)

**Table 5:** Median score and interquartile range (IQR) of QoL scales with regard to tumor stage (T1/2 and T3/4).

Tumor Stage	T1/T2 (*n* = 12)		T3/T4 (*n* = 14)	
Function Scale	Median	IQR	Median	IQR
Physical functioning I	67	(65–94)	42	(19–79)
Physical functioning II	100	(98–100)	92	(75–100)
Role functioning I	38	(25–100)	25	(6–50)
Role functioning II	63	(44–100)	50	(25–50)
Symptom scales				
Insomnia I	63	(19–100)	25	(25–94)
Insomnia II	63	(44–100)	50	(50–75)
Swallowing I	25	(22–55)	25	(13–34)
Swallowing II	72	(56–75)	38	(25–61)
Speech problems I	38	(19–81)	13	(0–25)
Speech problems II	50	(25–81)	25	(0–25)
Dry mouth I	75	(50–100)	50	(25–100)
Dry mouth II	75	(50–100)	38	(25–88)

Results marked with I are received by the answers of QU l, results marked with II are received by QU II.

**Table 6:** Median score and interquartile range (IQR) of quality of life (QoL) scales in a comparison of surgery with or without tracheotomy.

Tracheotomy	Yes		No	
	Median	IQR	Median	IQR
Insomnia I	25	(0–63)	50	(25–100)
Insomnia II	50	(25–63)	75	(50–100)
Swallowing I	25	(13–25)	25	(25–38)
Swallowing II	50	(25–72)	50	(50–69)
Speech problems I	25	(0–38)	25	(25–50)
Speech problems II	25	(25–50)	25	(25–50)

Results marked with I are received by the answers of QU l, results marked with II are received by QU II.

**Table 7:** Median score and interquartile range (IQR) of QoL scales in a comparison of gender.

Gender	Female		Male	
Symptom Scale	Median	IQR	Median	IQR
Insomnia I	25	(0–94)	63	(25–100)
Insomnia II	50	(31–75)	63	(50–100)
Trouble with social contact I	56	(50–94)	100	(75–100)
Trouble with social contact II	63	(50–94)	81	(59–100)

Results marked with I are received by the answers of QU l, results marked with II are received by QU II.

## Appendix A

Questionnaire 1 and 2

▪ These questions relate to your current state.
▪ Do you need help with?

1. Washing
1 very much 2 quite a bit 3 moderate 4 a little 5 not at all
2. Dressing
1 very much 2 quite a bit 3 moderate 4 a little 5 not at all
3. Eating
1 very much 2 quite a bit 3 moderate 4 a little 5 not at all
4. Do you feel limited in any way?
1 very much 2 quite a bit 3 moderately 4 a little 5 not at all
5. Were you short of breath?
1 very much 2 quite a bit 3 moderately 4 a little 5 not at all
6. Have you experienced any pain?
1 very much 2 quite a bit 3 moderate 4 a little 5 not at all
7. Current pain medication:
8. Is this medication sufficient?
9. Have you had trouble sleeping?
1 very much 2 quite a bit 3 moderate 4 a little 5 not at all
10. Are you tired during the day?
1 very much 2 quite a bit 3 moderately 4 a little 5 not at all
11. Have you had difficulty in concentrating on things, such as reading a newspaper or watching television?
1 very much 2 quite a bit 3 moderate 4 a little 5 not at all
12. Have you had difficulty remembering things?
1 very much 2 quite a bit 3 moderate 4 a little 5 not at all
13. Have you experienced a lack of appetite?
1 very much 2 quite a bit 3 moderate 4 a little 5 not at all
14. Have you experienced nausea?
1 very much 2 quite a bit 3 moderate 4 a little 5 not at all
15. Have you vomited at all?
1 very much 2 quite a bit 3 moderately 4 a little 5 not at all
16. Have you been constipated?
1 very much 2 quite a bit 3 moderately 4 a little 5 not at all
17. Have you had diarrhoea?
1 very much 2 quite a bit 3 moderate 4 a little 5 not at all
18. Do you feel frightened?
1 very much 2 quite a bit 3 moderately 4 a little 5 not at all
19. Do you worry?
1 very much 2 quite a bit 3 moderately 4 a little 5 not at all
20. Do you feel tense?
1 very much 2 quite a bit 3 moderately 4 a little 5 not at all
21. Do you have pain in your mouth?
1 very much 2 quite a bit 3 moderate 4 a little 5 not at all
22. Do you have soreness in your mouth?
1 very much 2 quite a bit 3 moderate 4 a little 5 not at all
23. Do you have a painful throat?
1 very much 2 quite a bit 3 moderate 4 a little 5 not at all
24. Do you have problems swallowing liquids?
1 very much 2 quite a bit 3 moderate 4 a little 5 not at all
25. Do you have problems swallowing pureed food?
1 very much 2 quite a bit 3 moderate 4 a little 5 not at all
26. Do you have problems swallowing solid food?
1 very much 2 quite a bit 3 moderate 4 a little 5 not at all
27. Have you choked when swallowing?
1 very much 2 quite a bit 3 sometimes 4 a little 5 not at all
28. Do you have a dry mouth?
1 very much 2 quite a bit 3 moderate 4 a little 5 not at all
29. Do you have problems with your sense of smell?
1 very much 2 quite a bit 3 moderate 4 a little 5 not at all
30. Do you have problems with your sense of taste?
1 very much 2 quite a bit 3 moderate 4 a little 5 not at all
31. Has your appearance bothered you?
1 very much 2 quite a bit 3 moderately 4 a little 5 not at all
32. Do you have trouble talking to other people?
1 very much 2 quite a bit 3 moderate 4 a little 5 not at all
33. Do you have trouble when receiving visitors?
1 very much 2 quite a bit 3 moderate 4 a little 5 not at all
34. Have you lost weight?
1 very much 2 quite a bit 3 moderately 4 a little 5 not at all
35. Have you gained weight?
1 very much 2 quite a bit 3 moderately 4 a little 5 not at all
